# Anticoagulation in Neonatal ECMO: An Enigma Despite a Lot of Effort!

**DOI:** 10.3389/fped.2019.00366

**Published:** 2019-09-13

**Authors:** Katherine Cashen, Kathleen Meert, Heidi Dalton

**Affiliations:** ^1^Division of Critical Care Medicine, Department of Pediatrics, Children's Hospital of Michigan, Wayne State University, Detroit, MI, United States; ^2^Division of Critical Care Medicine, Department of Pediatrics, INOVA Heart and Vascular Institute, Inova Fairfax Medical Institute, Falls Church, VA, United States; ^3^Department of Surgery, George Washington University, Washington, DC, United States

**Keywords:** extracorporeal membrane oxygenation, neonate, anticoagulation, hemostasis, monitoring, thrombosis, bleeding

## Abstract

Extracorporeal membrane oxygenation (ECMO) is a valuable modality used to support neonates, children, and adults with cardiorespiratory failure refractory to conventional therapy. It requires use of anticoagulation to prevent clotting in the extracorporeal circuit. Balancing bleeding from excessive anticoagulation with thrombotic risk remains a difficult aspect of ECMO care. Despite many advances in ECMO technology, better understanding of the coagulation cascade and new monitoring schemes to adjust anticoagulation, bleeding and thrombosis remain the most frequent complications in ECMO and are associated with morbidity and mortality. In neonates, ECMO is also complicated by the immature hemostatic system, laboratory testing norms which are not specific for neonates, lack of uniformity in management, and paucity of high-quality evidence to determine best practices. Traditional anticoagulation focuses on the use of unfractionated heparin. Direct thrombin inhibitors are also used but have not been well-studied in the neonatal ECMO population. Anticoagulation monitoring is complex and currently available assays do not take into account thrombin generation or platelet contribution to clot formation. Global assays may add valuable information to guide therapy. This review provides an overview of hemostatic alterations, anticoagulation, monitoring and management, novel anticoagulant use, and circuit modifications for neonatal ECMO. Future considerations are also presented.

## Introduction

Extracorporeal membrane oxygenation (ECMO) is a valuable modality used to support neonates, children, and adults with cardiorespiratory failure refractory to conventional therapy. Neonatal ECMO has evolved over the past 50 years with more than 41,700 neonates undergoing this lifesaving modality (1). Survival in the neonatal ECMO population remains highest with 73% survival to hospital discharge in the respiratory cohort and 42% survival to discharge in the cardiac cohort (1). However, despite improvements in ECMO technology and institutional experience, bleeding and thrombosis remain significant complications and are associated with worse outcome (2–4). The most recent Extracorporeal Life Support Organization (ELSO) registry report found that 11% of neonates placed on ECMO for respiratory and cardiac indications suffered from an intracranial hemorrhage 7–26% suffered surgical site bleeding, and 1–2% had gastrointestinal hemorrhage. Neonates with bleeding complications had significantly increased mortality compared to neonates without bleeding complications (2). The Bleeding and Thrombosis During ECMO (BATE) study performed by the Collaborative Pediatric Critical Care Research Network also found high rates of bleeding and thrombosis during neonatal ECMO. In the BATE study, 60–77% of neonates placed on ECMO for respiratory or cardiac indications suffered from bleeding events and 19–23% of these consisted of intracranial hemorrhage. Thrombotic events were recorded in 32–44% of the neonatal cohort. In the BATE study, 4–13% of these thrombotic events were patient-related (intracranial infarction, limb ischemia, aortopulmonary shunt clot, and other) and 25–40% were circuit-related. Both bleeding and thrombosis increased morbidity and mortality (3).

Titration of anticoagulation to limit bleeding and thrombosis remains challenging in neonates. The mechanisms of bleeding and thrombosis are complex and dynamic and involve multiple alterations in hemostatic factors. The developmental hemostatic system, increased risk for hemorrhage in the developing brain, heterogeneous disease processes that lead to initiation of ECMO, variability in anticoagulation strategies and lack of high-quality evidence to direct practice all contribute to the challenges in managing anticoagulation in the neonatal population. In this review, we provide an overview of developmental hemostasis, hemostatic alterations, anticoagulation, monitoring, management, and novel anticoagulant use as well as circuit modifications for neonatal ECMO.

## Developmental Hemostasis

Hemostatic equilibrium involves both procoagulant and anticoagulant factors and evolves from fetal to adult life (5–9). Primary hemostasis which is dependent on platelet adhesion, activation, and aggregation is different in the neonatal period compared to the rest of childhood and adulthood. The platelet count of neonates is usually normal or elevated but platelet hyporeactivity is well-described (10–13). However, despite hyporeactive platelets the bleeding time and platelet closure time (a measure of platelet function) are shortened in neonates and do not normalize until the first month of life (11, 12). Higher levels of von Willebrand factor (VWF) and higher percentage of larger VWF multimers likely increase the adhesive activity of platelets in neonates despite the overall platelet hyporeactivity (11, 13, 14). Thus, in healthy neonates elevated VWF balances platelet hyporeactivity and normal hemostasis is maintained.

Secondary hemostasis which consists of the coagulation cascade and ultimately leads to the formation of fibrin factors is substantially different in neonates than adults. At birth, the plasma levels of most coagulation proteins are around half of those measured in adults. Prolonged prothrombin time (PT) and partial thromboplastin time (PTT) in neonates has been consistently reported (8, 9). Decreased anticoagulant factors including protein C and S, and antithrombin as well as decreased thrombin generation and reduced clot lysis have been reported in neonates (9, 15). In healthy neonates hemostatic equilibrium is maintained but in critically ill neonates on ECMO lack of reserve capacity and immaturity of the coagulation system interferes with this fine balance and disequilibrium with resultant bleeding or thrombosis is common.

## Hemostatic Alterations During ECMO—ECMO Induced Coagulopathy

### The ECMO Circuit

**Figure 1:**
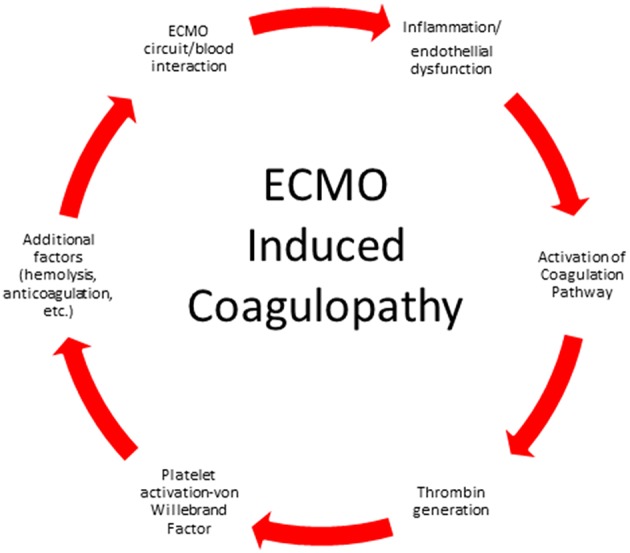
Factors contributing to ECMO induced coagulopathy.

The ECMO circuit consists of a mechanical blood pump, gas exchange devices, heat exchanger, tubing and cannulas. When blood is exposed to the non-biologic surfaces of the circuit, activation of the coagulation pathway and inflammatory response pathway occur (Figure 1). Exposure of blood to the non-endothelial surface of the extracorporeal tubing activates platelets, factor XII and kallikrein-kinin system, tissue factor, and von Willebrand factor, fibrinolysis and inflammation (16–20). Turbulent flow and shear stress from the pump, tubing, and cannula contributes to cellular damage and platelet activation. The continuous exposure of the blood to the non-endothelial surface shifts the normal hemostatic balance to a hypercoagulable state and requires systemic anticoagulation.

Our current understanding of the biomaterial interaction of the ECMO circuit and patient's blood involves surface contact and subsequent hemostatic activation. Fibrinogen binds to the ECMO circuit non-endothelial surfaces within minutes of contact followed by binding of coagulation factors, cellular adhesion and activation of platelets and polymorphonuclear (PMN) cells (21). Exposure of tissue factor and activation of circulating Factor VII then causes downstream thrombin generation. Activation of the complement system also leads to further activation of platelets and PMNs and increased adhesion and release of cytokines contributing to the pro-inflammatory hypercoagulable state (20–24).

To add even more complexity to the blood surface interaction some reports suggest distinct periods of activation in neonates. In the first 24 h of ECMO, contact activation and complement activation occur similar to the pattern of activation seen in cardiopulmonary bypass. A second period of activation was observed 72 h after EMCO initiation and was characterized by clotting and fibrinolytic activity without activation of the complement system (25). Various strategies have been used to mitigate the contact and hemostatic activation of the ECMO surface and will be detailed in the management section.

### Underlying Disease, Endothelial Dysfunction and Inflammation

The underlying disease process that leads to ECMO initiation plays an important role in ECMO induced coagulopathy. Neonates with a primary respiratory diagnosis such as meconium aspiration syndrome likely have very different coagulation profiles than neonates with sepsis or cardiac surgical patients who have undergone cardiopulmonary bypass. Thrombocytopenia also varies based on the pre-existing disease and platelet count prior to cannulation and may contribute to bleeding risk (26). On the other hand, continuous activation of the coagulation system during ECMO may lead to ongoing consumption of platelets and coagulation factors and contribute to a prothrombotic state.

Endothelial dysfunction during neonatal ECMO is also due to both the underlying disease process leading to ECMO initiation and the ECMO circuit itself (27–29). Damaged endothelium via expression of tissue factor activates procoagulant factors and contributes to ECMO induced coagulopathy (28–31). Critically ill neonates often have a generalized inflammatory response and associated coagulopathy. Underlying illness may lead to a hypercoagulable state or a hypocoagulable state and contributes to immune dysfunction and endothelial dysfunction and subsequent consumption of hemostatic factors (32–34). Many of the molecular mechanisms by which inflammation contributes to excessive activation of the coagulation cascade have been delineated and the cross-talk between innate inflammation and coagulation is well-described (35). This cross-talk between the systems may lead to unopposed amplification of the coagulation cascade in the setting of ongoing inflammation and may result in a hypercoagulable state and contribute to tissue damage and thrombosis.

### Thrombin Generation

Thrombin is a key enzyme in coagulation that converts fibrinogen to fibrin, activates factor XIII, factor V, factor VIII, factor XI and activates platelets. Thrombin generation also occurs when endothelial injury causes exposure of tissue factor to factor VII (30). Thrombin has a large array of functions and formation occurs during different stages of hemostasis. Anticoagulation with unfractionated heparin (UFH) prevents clot formation but does not stop thrombin generation or coagulation within the circuit (36). Neonates show a persistent increase in thrombin generation and fibrinolysis activation despite anticoagulation in a distinctly different pattern than is seen with children and adults (37).

### Platelet Activation and Von Willebrand Factor

Platelets adhere to the non-endothelial surfaces of the circuit and react with other activated components of the coagulation and complement systems increasing the risk of thrombotic complications. Both quantitative and qualitative platelet dysfunction has been described during ECMO and is associated with bleeding and mortality (31, 38) Thrombocytopenia is common during ECMO and more pronounced in neonates than older children (26). Severe thrombocytopenia from platelet consumption may lead to ongoing platelet transfusion, associated multiple organ dysfunction, microthrombi formation and immune dysregulation (39, 40). Platelets are also activated by shear stress from flow through the ECMO cannulas which is associated with decreased expression of platelet adhesion and structural molecules (41). Platelet microparticles, small circulating fragments of platelet plasma membranes, are produced by platelets during periods of shear stress and participate in thrombus formation (42). Platelet microparticles are increased in neonatal ECMO systems *in vivo* but no study to date has demonstrated that platelet microparticles contribute to a prothrombotic state *in vitro* (43).

Von Willebrand Factor (VWF) is a plasma glycoprotein that binds to FVIII, platelet surface glycoproteins and connective tissue. VWF forms a complex with FVIII that protects FVIII from degradation by activated protein C and localizes FVIII to sites of platelet plug and clot formation (44). Acquired VWF syndrome occurs during ECMO due to a loss of high molecular weight VWF multimers from shear stress. Disrupting VWF multimers is associated with increased bleeding complications (45, 46).

### Additional Considerations

#### Hemolysis

Hemolysis measured by plasma free hemoglobin levels has been associated with increased morbidity and mortality during ECMO (47, 48). Increased hemolysis has been reported in neonates compared to older children likely due to increased shear stress from flow through smaller caliber cannulas, increased fetal red blood cells which show greater susceptibility to mechanical stress than adult red blood cells, and higher hemoglobin concentration in neonates with increased blood viscosity (47, 48). Of note, measurement of plasma free hemoglobin is not uniform across centers. When measured, hemolysis (plasma free hemoglobin >50 mg/dL) was present in over 50% of patients and was associated with need for subsequent ECMO component change within 3 days (3).

## Anticoagulation Strategies

### Unfractionated Heparin

Unfractionated heparin (UFH) remains the most commonly used anticoagulation agent during ECMO (49). UFH potentiates (up to 1,000 times) the anticoagulant effect of antithrombin III (ATIII) by forming a UFH-ATIII complex that inactivates free thrombin and prevents further thrombin generation. UFH also weakly inhibits factor Xa (50). UFH binds to endogenous plasma proteins or heparin binding proteins including platelet factor 4 and high molecular weight multimers of VWF (51). The advantages of UFH include its low cost, short half-life, reversibility, and familiarity with use. Other advantages of UFH are its non-anticoagulant effects. Heparin has anti-inflammatory properties, inhibits reactive oxygen species generation, has tissue repair and protection properties and cardiovascular protective effects (52, 53). While most ECMO research focuses on limiting heparin exposure, higher heparin infusion dose was associated with decreased daily plasma free hemoglobin levels in neonates and children and improved survival in several studies (47, 54). UFH protocols usually include a bolus loading dose of UFH (50–100 units/kg) followed by an UFH infusion (10–51 units/kg/h) titrated for activating clotting time (ACT)or Anti-factor Xa assay activity (54). But, again the major benefit of UFH is the rapid reversibility.

The disadvantages of UFH include pharmacokinetic alterations depending on disease severity, renal failure, and increased volume of distribution in neonates (55).Variation in patient response to fixed dosing in part due to heparin binding proteins and the subsequent reduced anticoagulant activity of UFH especially in the neonatal population. As heparin requires a specific polysaccharide sequence to bind to ATIII, variability in activity between manufactured batches also exists and may help explain the individual responses noted. Another problem is heparin resistance related to reliance on ATIII levels which are lower in critically ill children and neonates and UFH's inability to inhibit factor Xa bound to platelets (56). Evidence suggests that ATIII activity often decreases over time in children on ECMO (57–60). Many centers routinely replace ATIII in an attempt to maximize heparin effect. ATIII can be supplemented by transfusion of fresh frozen plasma or by infusing ATIII concentrates. But, ATIII concentrates are expensive and studies in the neonatal and pediatric population have found mixed results when ATIII supplementation is utilized (57–60). A recent large observational report (including 5,360 neonates) found that ECMO patients supplemented with ATIII had an increased number of thrombotic and hemorrhagic events and longer hospital length of stay with no difference in mortality (61). A limitation of this study was that the rate of complications was not adjusted for exposure or illness severity. At this time, clear recommendations and indications for ATIII supplementation are lacking and use should be judicious.

Another potential disadvantage of UFH is development of heparin induced thrombocytopenia (HIT), an immune mediated adverse drug reaction caused by antibodies to complexes of platelet factor 4 and heparin. The risk of thrombotic events and associated morbidity and mortality is high. A recent systematic review on HIT in children found seroconversion in 0–1.7% of neonates but no cases of neonatal HIT. This could be due to challenges in defining HIT in the neonatal population, lack of studies in this population, or that HIT is indeed rare in neonates. Thus, while this is a disadvantage of heparin use in adults and pediatric patients it should not be a major concern for neonatal ECMO (62).

### Direct Thrombin Inhibitors

Direct thrombin inhibitors have been rarely used in neonatal ECMO patients. Direct thrombin inhibitors such as bivalrudin, argatroban, and lepirudin directly bind to active sites on thrombin providing a greater reduction in thrombin compared to UFH. Direct thrombin inhibitors inhibit both free and bound thrombin, are ATIII independent, are not inhibited by platelet factor 4, and have more predictable dose effects because they do not bind to plasma proteins (63). Use of direct thrombin inhibitors are currently limited in children and reserved for those with allergy to UFH, HIT, and heparin resistance due to cost, safety and dosing concerns (31).

Of the direct thrombin inhibitors, bivalrudin has been utilized the most in the pediatric population but isolated reports of argatroban and lepirudin have been published. A handful of reports describe argatroban use in pediatric ECMO patients with HIT and are associated with good outcome (64–67). A prospective study of argatroban use in pediatrics as an alternative to UFH enrolled 18 pediatric patients and included 2 pediatric ECMO patients. Both of these patients had HIT and were treated with an initial bolus dose of 100 μg/kg followed by initial infusion of 2 μg/kg/min to reach a target ACT 180-220 (68). One of these patients died from progressive thrombosis and severe cardiac dysfunction during cardiac transplantation. In this report, argatroban in pediatric patients was used to achieve aPTT levels 1.5 to 3 times baseline with rapid achievement of therapeutic values however enrollment was limited, and complications persisted (68). Successful administration of lepirudin to treat HIT during ECMO has been reported in a 21-month-old child but two other children (a 15-year-old and a 4-year-old) ultimately died (69–71). In these studies, a lepirudin bolus dose was used ranging from 0.1 to 0.4 mg/kg loading dose followed by an infusion of 0.12 mg/kg/h and titrated in 0.01 mg/kg/h increments. Target aPTT levels were 1.5–2.5 times baseline values. Availability of lepirudin is currently limited.

The shorter half-life of bivalrudin (~25 min) makes this agent more attractive compared to the longer half-life of argatroban (40 min) and lepirudin (78 min). Half-life may be increased in renal failure as roughly 20% is renally cleared while the remainder is degraded by proteases. Neonates compared to older children have more rapid clearance of bivalrudin and a lower average serum concentration than older children (72). Bivalrudin use and target aPTT and dosing is variable in reported case series of pediatric ECMO patients (72–74). Pediatric reports have used either no bolus or a small bolus of 0.5 mg/kg loading dose followed by an infusion of 0.05–0.15 mg/kg/h and targeting aPTT 1.5–2 times baseline (74–76). Others report a lower bolus 0.05–0.5 mg/kg loading dose followed by an infusion rate of 0.03–0.3 mg/kg/h and targeting aPTT 1.5–2.5 times baseline (54, 76). Ranucci and colleagues used bivalrudin in post-cardiotomy pediatric ECMO patients and reported less total blood loss and decreased transfusion needs (75). Direct thrombin inhibitor use is becoming standard in some centers, although little complete data on safety and efficacy is available in the literature except for single site reports.

Unlike UFH, direct thrombin inhibitors do not provide inhibition to the contact pathway which contributes to thrombus formation on ECMO (77). Safety and dosing concerns as well as lack of reversibility make direct thrombin inhibitors less attractive in the neonatal ECMO population as a first-line agent without additional systematic study.

### Antiplatelet Agents

Platelet activation on ECMO is well-described however use of antiplatelet agents (aspirin, dipyridamole, clopidogrel) is rare (49). Experience from pediatric ventricular assist device (VAD) patients suggests that there may be utility in adding antiplatelet agents for anticoagulation. In the Berlin EXCOR Investigational Device Exemption trial the guideline for anticoagulation included use of aspirin, dipyridamole, and enoxaparin or warfarin (78). Although still substantial, pediatric VAD patients have decreased bleeding complications compared to pediatric ECMO patients and direct treatment comparisons to the neonatal ECMO population should not be made. In addition, thrombocytopenia is more pronounced in the neonatal ECMO population compared to older children. Neonates are at the highest risk for intracranial hemorrhage therefore antiplatelet agents are rarely used. This is another area in need of specific evaluation. Antihemostatic medications are shown in Table 1.

**Table 1:** Antihemostatic agents. ATIII is antithrombin III, HIT is heparin induced thrombocytopenia, cAMP is cyclic adenosine monophosphate, ADP is adenosine diphosphate.

**Drug**	**Mechanism of action**	**Onset of action**	**Half-life**	**Excretion**	**Pro**	**Con**
Unfractionated Heparin	Potentiates the action of antithrombin III and inactivates thrombin (inactivates factors IXa, Xa, XIa, XIIa, and plasmin) and prevents the conversion of fibrinogen to fibrin	Immediate	Dose and age dependent: median 1.5 h, shorter in premature neonates	Renal, at therapeutic doses elimination occurs rapidly via non-renal mechanisms	Low cost, short half life, reversible	Variable patient response, variablility in activity, reliance on ATIII, HIT
**DIRECT THROMBIN INHIBITORS**						
Bivalrudin	Direct thrombin inhibitor	Immediate	25 min	Proteolysis 75–80%, Renal 20–25%	Not dependent on ATIII, inhibits free and bound thrombin, predictable dose effects, Used in HIT	No antidote, no inhibition to contact pathway
Argatroban		Immediate	39–51 min	Hepatic		No antidote, no inhibition to contact pathway
Lepirudin		Immediate	80 min	Renal		No antidote, no inhibition to contact pathway, unavailable
**ANTIPLATELET AGENTS**						
Aspirin	Irreversibly inhibits cyclooxygenase-1 and 2 enzymes which results in decreased formation of prostaglandin precursors and inhibition of thromboxane A2	Immediate release, non-enteric coated platelet inhibition within 1 h	Oral: Plasma concentration 15–20 min, 3 h at lower doses	Renal	Familiarity of use	Gastritis, normal platelet function only returns when new platelets are released, Reye syndrome with prolonged high dose aspirin
Dipyridamole	Inhibits the uptake and metabolism of adenosine in platelets, endothelial cells and erythrocytes, inhibits platelet cAMP	Peak plasma concentrations ~2 h	Oral tablets: Biphasic; initial half life 40–80 min and terminal half life 10–12 h.	Hepatic	Mediates coronary vasodilation	Variable absorption from gastrointestinal tract, headache, vasodilation
Clopidogrel	Irreversible blockade of the ADP receptor on the platelet surface	Dose dependent; 300–600 mg loading dose onset within 2 h, smaller doses within second day of treatment	6-8 h	Hepatic		Bleeding, decrease in white blood cell count, irreversibly inhibits platelet aggregation, normal platelet function only returns when new platelets are released

### Circuit Modifications

In an attempt to mitigate the bleeding and thrombotic effects of ECMO, circuit modifications have been developed including surface modifications. Heparin-coated circuits are associated with reduced platelet, leukocyte and coagulation activation and decreased thrombin generation (79). Heparin-coated circuits were used by 59% of ECMO center respondents in a multicenter survey of ELSO centers (49). Nitric oxide embedded surfaces are also under development and are associated with local antiplatelet properties preventing platelet adhesion (80). Finally, modifications to the membrane oxygenator material and coating have also been made to decrease thrombotic risk. Newer generation oxygenators are made with polymethylpentene or polypropylene hollow fibers which are hydrophobic and allow gas exchange without allowing blood or protein absorption (80). The efficacy of surface coatings during prolonged ECMO runs is also unknown.

## Anticoagulation Monitoring

Anticoagulation monitoring is extremely variable and remains controversial in ECMO patients (49). Assessing coagulation in critically ill neonates is complex and limited by inability to standardize testing across laboratories. Center specific anticoagulation protocols have been developed with mixed results and the ideal monitoring tool is unclear. Almost 97% of centers reported using the ACT with many centers sending additional anticoagulation testing. There is no widely accepted gold standard in anticoagulation and tests developed *in vitro* may not always represent *in vivo* phenomena. While anticoagulation monitoring is complex, the number of tests and frequency of testing should be determined based on the individual patient's needs and expertise at each center.

### Activated Clotting Time (ACT) Values and Limitations

The ACT is available in real time and measures the time for whole blood to clot when activated by kaolin, celite or glass beads. ACT is a global functional test of hemostasis and is low cost. ACT is prolonged with anticoagulant use but can also be prolonged by hemodilution, hypothermia, decreased coagulation factor levels, elevated d-dimer, hypofibrinogenemia and thrombocytopenia. These conditions could overestimate heparin effect (16, 31). ELSO guidelines suggest a target ACT of 180–220 in uncomplicated ECMO patients.

### Activated Partial Thromboplastin Time (APTT)

The aPTT is the time for recalcified, citrated, platelet poor plasma to clot when activated with an intrinsic pathway activator. Baseline aPTT is prolonged in neonates and aPTT levels are affected by coagulation factor deficiency, hyperbilirubinemia, hyperlipidemia, anti-phospholipid antibodies and elevated C reactive protein. Variances in laboratory methods for measurement between sites also complicates aPTT interpretation.

### Anti-factor Xa Concentration Assay

Anti-Xa assay measures the inhibition of factor Xa by heparin in plasma. This test is more specific to the assessment of heparin effect because it is not affected by other coagulation proteins or platelets. Anti-Xa measures the UFH-ATIII complex levels not the UFH concentration. Some laboratories add ATIII to their Anti-Xa assays to normalize the ATIII levels. In neonatal patients with lower ATIII the addition of ATIII to the assay can significantly impact the results. So, for neonates Anti-Xa assays without added ATIII are preferable. However, levels can be affected by elevated hemoglobin level, plasma free hemoglobin, lipids, and bilirubin. More centers are using Anti-Xa assays as part of their anticoagulation protocols and most target levels 0.3-0.7 IU/mL (54). Anti-Xa assay has a better correlation to UFH dose than ACT to UFH dose (81). But, while Anti-Xa assays may provide advantages over other laboratory testing this value alone cannot be used to determine hemostatic potential and a multifactorial approach is needed.

### TEG or ROTEM Guided Algorithm

**Figure 2:**
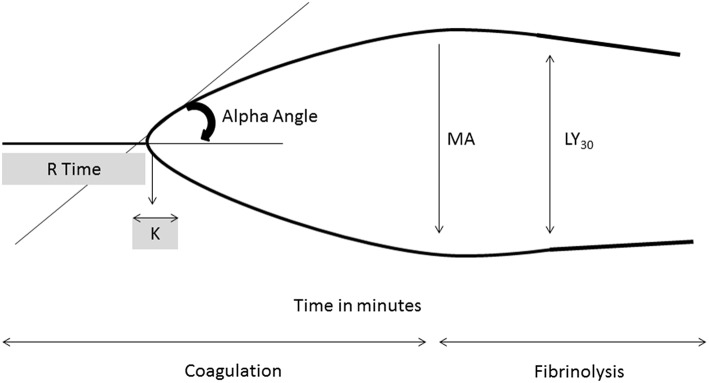
Thromboelastography tracing with key parameters. R time, time of latency from start of test to initial fibrin formation; K, time taken to achieve a certain level of clot strength (amplitude of 20 mm); α angle (degrees), measures speed at which fibrin build up and cross linking takes place, rate of clot formation; MA, represents the strength of the fibrin clot; LY30 (%), percentage decrease in amplitude at 30 min post MA and gives measure of degree of fibrinolysis.

Global assays of coagulation measure the viscoelastic properties of blood and can provide information on clot dynamics and fibrinolysis. Like the ACT, thromboelastography (TEG) and rotational thromboelastography (ROTEM) can be performed in real time which would be a major advantage. Unfortunately, many centers do not have these capabilities. These assays use an activator Kaolin or tissue factor and are affected by thrombocytopenia and coagulation factor deficiencies. TEG can detect deficiencies in hemostasis throughout the coagulation cascade and provide information regarding fibrinolysis. The measurements of TEG can provide information about initial clot formation (R time), clot acceleration (α angle), maximum clot strength (MA), and fibrinolysis (Ly30) (Figure 2). Therapeutic ranges in the neonatal population are not well-established but recent studies have attempted to determine optimal values to minimize bleeding and thrombosis (82).

### Novel Global Assays

Novel global assays of coagulation may have an application in the neonatal ECMO population. Clot formation and lysis assay (CloFAL) a global measure of fibrinolysis, global fibrinolytic capacity (GFC) in whole blood, and the thrombin generation assay (measured by calibrated automated thrombogram-CAT) are all novel assays that have been applied in the research setting and have not been applied clinically to the ECMO population. The CAT seems promising because this plasma-based assay can be performed with and without platelets and may give important information about thrombin generation and risk of bleeding and thrombosis.

Different combinations of laboratory monitoring have been proposed with mixed results. Initial reports suggested decreased blood product use, decreased hemorrhagic complications, and increased circuit life in a pediatric population of ECMO patients after initiation of a comprehensive monitoring protocol (83). However, others have reported no difference in outcomes when comparing a complicated vs. simple monitoring strategy (84). In the era of increasingly complicated patients, using only one anticoagulation laboratory test is likely ill-advised but the frequency of testing and ideal combination of anticoagulation monitoring tests is still unclear in the neonatal ECMO population. The impact of iatrogenic blood loss is especially concerning in the neonatal population and evidence comparing the different anticoagulation measurements with the risk of blood loss is lacking. Use of one measure with whole blood and one with plasma effect may seem a reasonable approach.

## Management

### Bleeding

Bleeding events occurred in 77% of the neonatal cardiac patients and 60% of the neonatal respiratory patients in the BATE study and were associated with increased mortality (3). Management of bleeding depends on the site of bleeding and primary cause. Initial management includes decreasing anticoagulant dose and using blood product replacement based on known deficiencies (i.e., platelet transfusion for thrombocytopenia). Target levels for hemoglobin, platelets, and fibrinogen may be changed in the setting of bleeding. In addition, acquired VWF deficiency and FXIII deficiency should be considered in bleeding patients with adequate fibrinogen and platelet counts. Cessation of anticoagulant use (mainly heparin) has also been employed, but the lower ECMO flow rates in neonates may make risk of circuit thrombosis higher than in older children and adults.

Antifibrinolytic agents like aminocaproic acid and tranexamic acid have been used to manage surgical site bleeding in pediatric patients. In pediatric ECMO patients, aminocaproic acid was associated with decreased incidence of surgical bleeding without an increase in thrombotic events (84). Tranexamic acid use was associated with decreased postoperative blood loss in infants with congenital diaphragmatic hernia repair while on ECMO (85).

Recombinant activated Factor VII (rVIIa) and Prothrombin Complex Concentrate (PCC) have been used to treat severe refractory bleeding on ECMO in adult and pediatric patients. rVIIa forms complexes with tissue factor and binds to platelet surfaces to generate thrombin and is given in doses of 40–90 ug/kg intravenously and may be repeated every 1–4 h in repeated doses (54). Reports of decreased bleeding and reduced need for transfusion must be balanced with reports of fatal thrombosis (86–88). PCC contains factor II, VII, IX, and X and some contain protein C and S and doses of 25–50 IU/kg have been used in pediatric cardiac surgical patients (89). An adult case report describes fatal circuit thrombosis when a man was treated with rVIIa and PCC for refractory bleeding (90). rVIIa and PCC needs additional study for treatment of severe refractory bleeding during ECMO before recommendations can be made in neonates.

### Thrombosis

Thrombotic events were recorded in 32% of neonates on ECMO for respiratory indications and 44% of neonates on ECMO for cardiac indications in the BATE study (3). Thrombus formation, reportedly occurs during periods of low ECMO flow, at sites of stasis or turbulent flow, and during periods of inadequate anticoagulation (56). Thus, management first involves avoiding these states to prevent thrombosis. If thrombus formation has already occurred, then changing of circuit components may be necessary. Anticoagulation targets may be modified and if thrombotic events are ongoing or HIT is suspected then an alternate anticoagulant should be considered.

### Transfusion Thresholds

The optimal threshold for transfusion of packed red blood cells, platelets, fresh frozen plasma, and cryoprecipitate are unknown with a paucity of data to guide clinical decision-making. Thresholds vary by center, location of ECMO care, and by clinical scenario. Multiple reports suggest that increased transfusion volume is associated with increased mortality (91, 92). Platelet transfusion thresholds are variable and volume of platelet transfusion is associated with mortality (26). While most centers focus on platelet count as the transfusion trigger, there is little to no data on platelet activity and associated platelet count in neonatal ECMO. Studies evaluating such associations and evaluating restrictive transfusion strategies are needed to guide therapy.

## Future Steps

### Additional Circuit Modifications

Research to develop fluid-repellent surfaces has been ongoing. This technology has been used to coat medical devices in the laboratory and in an animal model but has not been applied to the clinical setting (79). Endothelialization of ECMO surfaces via different techniques is another promising strategy currently being studied to inhibit thrombogenesis (79). Development of ECMO circuit modifications to prevent thrombosis and avoid bleeding associated with systemic anticoagulation could prevent many of the complications seen during ECMO.

### Targeted Animal Studies

Factor XII-mediated activation and coagulation may contribute to thrombosis with biomaterial contact (92). Two studies in an animal model suggest that factor XII inhibition (via a monoclonal anti-factor XIIa antibody and a FXII inhibitor) decrease arterial and venous thrombus formation on ECMO but do not increase the rate of bleeding. Thus, this targeted therapy could decrease thrombotic risk without increased risk of bleeding and avoidance of systemic anticoagulation (92, 93).

### Phenotyping and Genomics

One of the most perplexing aspects of ECMO is the fact that some patients with similar profiles, ECMO equipment, anticoagulation protocols and monitoring results bleed while others clot and others have neither complication. These facts suggest that individual variability may be important aspects in eliminating thrombotic risk and events. Genetic testing and variation in clotting and bleeding risk has been well-reported in multiple disease processes. In 1993, small endogenous nucleotides which were termed microRNAs (miRNAs) were identified as post-transcriptional regulators of gene expression. Further investigations have demonstrated that miRNAs are regulators of many biologic processes, including hemostatic function (94). miRNAs have been identified which affect many hemostatic factors in the coagulation cascade such as protein C and S production, tissue factor, platelets, fibrinogen and others. They have been shown to be associated with conditions of thrombosis, such as stroke and ischemic heart disease. Platelets, adams13, tissue factor, fibrinogen and proteins regulating fibrinolysis have all been suggested as important players in hemostasis during ECMO (95). The changes in miRNA production related to the hemostatic system on exposure to ECMO has not been evaluated, but establishing what changes in miRNA occur with ECMO may provide new data to develop these as biomarkers for thrombotic risk or even therapeutic intervention. The ability to determine what patients are most at risk for thrombosis may help tailor anticoagulation management, decrease or eliminate need for anticoagulation and improve outcomes. While promising, the connection between genetic variation and phenotypic expression in critically ill neonates on ECMO is not well-understood and needs further study.

## Conclusion

Optimal anticoagulation in neonatal ECMO patients remains an enigma. Bleeding and thrombosis are common and involve multiple alterations in hemostatic factors. Neonatal anticoagulation is challenging due to the developmental hemostatic system, heterogeneous disease processes that lead to initiation of ECMO, variability in anticoagulation strategies and lack of high-quality evidence to direct practice. UFH remains the most commonly used systemic anticoagulant but there have been increasing reports of DTI use. Comprehensive assessments of hemostasis using more than one of the currently available assays (ACT, anti-Xa, PT, aPTT) is still variable across centers and suboptimal in isolation because these tests do not take thrombin generation or platelet contribution to clot formation into account. Global assays (TEG/ROTEM, CloFAL, CAT) may improve our anticoagulation management but still need to be studied in the neonatal population. A combination of whole blood and plasma assessment has also been suggested as optimal for anticoagulation management but no gold standard algorithm has been universally developed or accepted. Standard treatments for bleeding and thrombosis are lacking and case reports and expert opinion guides management. Optimal transfusion thresholds are unknown. Pragmatic multicenter trials randomizing neonates to restrictive vs. standard transfusion therapies are needed with well-defined endpoints. Detailed observational studies focused on sites with low neonatal rates of thrombosis and bleeding should be considered in order to compare anticoagulation algorithms. Then, with a protocol to standardize circuitry, transfusion thresholds, laboratory testing and clearly defined thrombotic and bleeding complications a trial to compare “best practice” anticoagulation strategy should be initiated. Finally, ongoing studies to develop new circuit modifications and targeted thrombotic factor inhibition are promising. Multicenter research focused on standardized anticoagulation protocols, monitoring and treatment are needed to improve our care of neonatal ECMO patients. Genomic investigation offers an exciting new area of research to achieve the “Holy Grail” of eliminating thrombotic risk and need for anticoagulation during extracorporeal support.

## Author Contributions

KC wrote the manuscript draft and finalized changes after revision and approval. KM and HD revised, read, and approved final manuscript.

### Conflict of Interest Statement

HD declares that she is the consultant medical director for Innovative ECMO Concepts. The remaining authors declare that the research was conducted in the absence of any commercial or financial relationships that could be construed as a potential conflict of interest.
